# Enhancing the Mechanical and Electrical Properties of Poly(Vinyl Chloride)-Based Conductive Nanocomposites by Zinc Oxide Nanorods

**DOI:** 10.3390/ma11112139

**Published:** 2018-10-30

**Authors:** Feng Qiu, Guangjian He, Mingyang Hao, Guizhen Zhang

**Affiliations:** National Engineering Research Center of Novel Equipment for Polymer Processing, Key Laboratory of Polymer Processing Engineering, Ministry of Education, School of Mechanical and Automotive Engineering, South China University of Technology, Guangzhou 510640, China; qiufeng868@gmail.com (F.Q.); hmyang1992@163.com (M.H.); guizhenzhang@scut.edu.cn (G.Z.)

**Keywords:** poly(vinyl chloride), zinc oxide nanorods, multi-walled carbon nanotubes-reduced graphene oxide hybrid nanoparticles, nanocomposites

## Abstract

A simple approach to decorate multi-walled carbon nanotube (MWCNT)–reduced graphene oxide (RGO) hybrid nanoparticles with zinc oxide (ZnO) nanorods is developed to improve the electrical and mechanical properties of poly(vinyl chloride) (PVC)/MWCNT–RGO composites. The ZnO nanorods act as “joint” in three-dimensional (3D) MWCNT–RGO networks and the hybrid particles strongly interact with PVC chains via p-π stacking, hydrogen bonds, and electrostatic interactions, which we confirmed by scanning electron microscopy (SEM) and Raman analysis. By introducing the ZnO nanorods, the RGO–ZnO–MWCNT hybrid particles increased 160% in capacitance compared with MWCNT–RGO hybrids. Moreover, the addition of RGO–ZnO–MWCNT to PVC resulted in the mechanical properties of PVC being enhanced by 30.8% for tensile strength and 60.9% for Young’s modulus at the loadings of 2.0 weight percent (wt.%) and 1.0 wt.%, respectively. Meanwhile, the electrical conductivity of PVC increased by 11 orders of magnitude, from 1 × 10^−15^ S/m to 1 × 10^−4^ S/m for MWCNT–ZnO–RGO loading at 5.0 wt.%.

## 1. Introduction

Polymeric nanocomposites, composed of polymers and nanofillers dispersed in a polymer matrix, have attracted the attention of researchers worldwide for their widespread application in the fields of chemistry, machinery, and optics [1,2,3]. In the past few decades, poly(vinyl chloride) (PVC), as a host polymer matrix in polymeric nanocomposites, caught much of the attention of global researchers due to its excellent chemical stability, bio-compatibility and low cost [4,5,6]. Based on PVC as a matrix of high-performance nanocomposites, the present challenge depends on the preparation of a satisfactory reinforcing filler, which can uniformly disperse and have strong interfacial interaction with the PVC matrix. Therefore, the need to find such a suitable filler in order to prepare high-performance nanocomposites is apparent.

Multi-walled carbon nanotubes (MWCNTs) are a prominent nanofiller to enhance the properties of polymer composites as a result of its unique combination of properties such as excellent chemical stability and excellent electrical, thermal and mechanical properties [7,8,9]. However, MWCNTs, due to their strong chemical inertness, make it difficult to disperse well in the polymer matrix, and their interface with the polymer matrix is very weak. Therefore, to obtain a better performance of the polymer material, the nanotubes are usually modified by either covalent or non-covalent methods [10]. Covalent functionalization involves either the direct attachment of functional groups to the graphitic surface or by linking functional groups which are inherent defects on the carbon nanotube’s (CNT’s) surface [10]. The functional groups, such as carboxyl and hydroxyl, can be introduced by chemical treatment, which can be used to either “graft from” or “graft to” polymers from the surface of CNTs [11]. Shadpour Mallakpour’s group improved the compatibility between PVC and MWCNTs through chemical treatment of carboxylated multi-walled carbon nanotubes (MWCNTs–COOH) with thiamine (Tm). This process achieved better CNT dispersion and enhanced mechanical properties of PVC-based nanocomposites [12,13,14,15]. Moreover, the combination of CNTs and graphene to improve the composite’s performance is an effective method. Zou [16] used carbon nanotubes/graphene composites to improve capacitive deionization performance. Graphene was employed to disperse carbon nanotubes in silicone rubber [17]. In combination, reduced graphene oxides with carbon nanotubes results in synergies with polypropylene nanocomposites [18]. However, the covalent chemical modification approach would destroy the sp^2^ hybridization of the CNTs, and thus affect their intrinsic electrical, thermal and mechanical properties so that they could not fully exert their ability to reinforce the polymer composite materials.

Without damaging the surface structure of the MWCNTs through covalent functionalization, the non-covalent bond approach is an effective method of well-dispersing MWCNTs in the polymer matrix [19]. CH–π is capable of forming stable CNT–polymer dispersions in many solvents due to the fact that the interaction sites between the carbon nanotubes and the macromolecules are large numbers [20]. Furthermore, aromatic compounds adsorbed to the surface of CNTs which employ π–π stacking were also used to improve compatibility between MWCNTs and polymers [21,22,23,24,25].

It is well known that single transition metal oxide nanoparticles, dissimilar from atomic and bulk counterparts, such as copper oxide (CuO) [26,27], zinc oxide (ZnO) [28], ferroferric oxide (Fe_3_O_4_) [29,30] and tantalum oxide (Co_3_O_4_) [31] exhibit special physical and chemical properties due to their large specific surface area and quantum size effects. In previous works, some researchers have demonstrated that adding metal oxides like ZnO particles to a PVC matrix can enhance the mechanical properties because of the strong interfacial interactions formed with PVC molecular chains. [32,33]. If these nanoparticles are present among MWCNTs, the strong attractive interactions between adjacent CNTs can be weakened, along with the overall specific surface area. Thus, ZnO might be able to help with the dispersion of MWCNTs and prevent their re-agglomeration in polymer [34].

In our work, we prepared MWCNT–ZnO–reduced graphene oxide (RGO) hybrid particle fillers, then used them to modify PVC for better mechanical and electrical performance. The precursor graphene oxide (GO) of RGO was used to disperse MWCNTs well in solution. Single ZnO nanorods interact strongly with MWCNTs and RGO through p–π stacking/electrostatic interaction. They uniformly disperse in three-dimensional (3D) networks, which consist of one-dimensional (1D) MWCNTs and two-dimensional (2D) RGOs similar to the “joints” in fishing nets, increasing the mechanical properties of the nanocomposites. Hydrogen bonds or electrostatic attraction interactions also exist between the ZnO and the PVC molecular chains. So, we prepared the MWCNT–ZnO–RGO hybrid particles that can be well-dispersed in the PVC matrix. With their strong interfacial interactions with the minimal addition of hybrid particles, the mechanical properties and electrical conductivity of the nanocomposite materials were substantially improved.

## 2. Experimental

### 2.1. Materials

The PVC was purchased from Sichuan Jinlu Resin Co. Ltd. (Deyang, China). The zinc nitrate hexahydrate (Zn (NO_3_)_2_·6H_2_O, 99.0%), sodium hydroxide (NaOH, 96.0%) and hydrazine hydrate (80.0% aq.) were obtained from Chengdu Kelong Chemical Reagent Factory (Chengdu, China) and Chongqing Chuandong Chemical Co. Ltd. (Chongqing, China), respectively. Graphene (layers < 40, size: 6–10 μm, thickness: 5–20 nm). The Tianjin Regent Chemical Co. Ltd. (Tianjin, China) offered ZnO. The multi-walled nanotubes (MWCNTs, TNIM4, average diameter: 10–30 nm, average length: 10–30 μm, purity: >95%) were bought from Chengdu Organic Chemical Co. Ltd. (Chengdu, China), Chinese Academy of Sciences.

**Figure 1:**
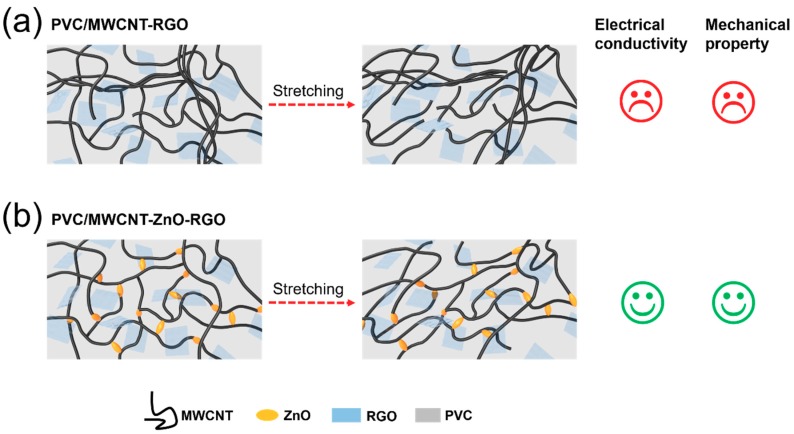
Scheme of multi-walled carbon nanotube (MWCNT)–reduced graphene oxide (RGO) particles (**a**) and MWCNT–zinc oxide (ZnO)–RGO particles (**b**) in a poly(vinyl chloride) (PVC) matrix before and after stretching.

We have designed the following schematic diagram (Figure 1) to describe the role of ZnO in the two composites: (a) the conductivity is poor when no zinc oxide nanorods are added into the PVC/RGO–MWCNT composites. When the composites are subjected to a stretching force, the hybrid particles are easily pulled apart in the matrix and exhibit poor mechanical properties. (b) When zinc oxide nanorods are added into the PVC/RGO–MWCNT composites, it can be seen that the ZnO nanorods act as joints to connect the RGO–MWCNT fillers and PVC chains; thus, the interaction between the hybrid particles and the matrix is improved. Consequently, under the action of a stretching force, the PVC/RGO–ZnO–MWCNT composites have better electrical conductivity and mechanical properties.

### 2.2. Synthesis of MWCNT–ZnO–RGO Hybrid Particles

The GO was prepared by the Hummers method [35]. Synthesis of MWCNT–ZnO–RGO hybrid particles: 225 mL of 1.0 mg/mL MWCNT aqueous solution and 75 mL of 1.0 mg/mL GO aqueous solution were added to a flask, then 0.073 g of 0.3 mol/L Zn(NO_3_)_2_ aqueous was added to the mixed solution. A diluted aqueous solution of sodium hydroxide was used to adjust the mixed solution to pH = 10–11 to form Zn(OH)_4_^2−^ particles [36]. Three milliliters of 80% hydrazine hydrate was added to the flask and then stirred at 100 °C for 24 h to form a yellow solution. After cooling, the solution was centrifuged to get the precipitate, which was then washed with deionized water and ethanol several times until the pH reached 7. The MWCNT–ZnO–RGO particles were obtained and dried. The synthetic procedure to obtain MWCNT–RGO hybrid particles was the same as MWCNT–ZnO–RGO, without the addition of the Zn(NO_3_)_2_ aqueous solution and sodium hydroxide.

### 2.3. Preparation of PVC/MWCNT–ZnO–RGO Nanocomposites

The PVC/MWCNT–ZnO–RGO nanocomposite films were prepared by the solution blending method. MWCNTs–ZnO–RGO hybrid particles (0.001 g) were dispersed in 50 mL tetrahydrofuran (THF) and sonicated for 1 h for later use. PVC powder (0.999 g) was added to THF, which was reheated and stirred until a transparent solution was obtained. Then, these two solutions were blended and ultrasonicated for half an hour. Lastly, the blended solution was poured onto the glass plates to evaporate the THF at 50 °C in an oven, which formed a film with an approximate thickness of 50 μm. PVC with 0.1 weight percent (wt.%), 0.2 wt.%, 0.5 wt.%, 1.0 wt.%, 2.0 wt.%, 3.0 wt.% and 5.0 wt.% MWCNT–ZnO–RGO filler content were employed to investigate the loading effect. As a control, the PVC/MWCNT–RGO composites were prepared with the same filler loadings. Table 1 shows all investigated nanocomposites.

### 2.4. Characterization

The structure and morphology of the MWCNT–ZnO–RGO and MWCNT–RGO hybrid particles were examined by field emission scanning electron microscopy (FE-SEM, S4800, Hitachi, Tokyo, Japan, at 10 kV) and X-ray diffraction (XRD) (Shimadzu XRD-7000, Kyoto, Japan, Cu Kα radiation, λ = 0.154 nm, 36kV and 20mA, 2*θ* = 6–70°, 4°/min^−1^). The Raman spectroscopy was recorded on a Renishaw InVia Reflex Raman Microscope (Wotton-under-Edge, UK) at excitation wavelengths of 633 nm. The electrochemical properties of electrochemical impedance spectroscopy (EIS) and cyclic voltammetry (CV) of the specimens were operated in a three-electrode cell by a CHI760e electrochemical workstation (CH Instruments, Austin, TX, USA).

The thermal analysis of the composites was tested by a differential scanning calorimetry device (NETZSCH DSC 200 F3 Maia, Selb, Germany) under nitrogen atmosphere with the process as follows: firstly, heated from 30 °C to 150 °C with a heating rate of 10 °C per minute, then maintained at 150 °C for 5 min to erase the thermal history; then, cooled down to 30 °C at the same rate of 10 °C per minute; lastly, heated to 150 °C at the same heating rate. The surface static contact angles of all specimens were examined by a contact angle device (JC2000C1, Shanghai Zhongchen Digital Technology Co., Ltd., Shanghai, China) at 23 ± 2 °C using deionized water and CH_2_I_2_. The electrical conductivity of the specimens (which used at least five specimens) was tested by a digital high resistance machine (PC68, Shanghai Precision Instrument Manufacture, Shanghai, China) at 23 ± 2 °C. For the mechanical properties of the composite films, the average value was obtained from at least five specimens at 23 ± 2 °C), and were measured by the tensile testing machine (INSTRON 5566, DatapointLabs, Ithaca, NY, USA) at a cross-head speed of 10 mm/min.

## 3. Results and Discussion

### 3.1. Characterization of the Nanoparticles

**Figure 2:**
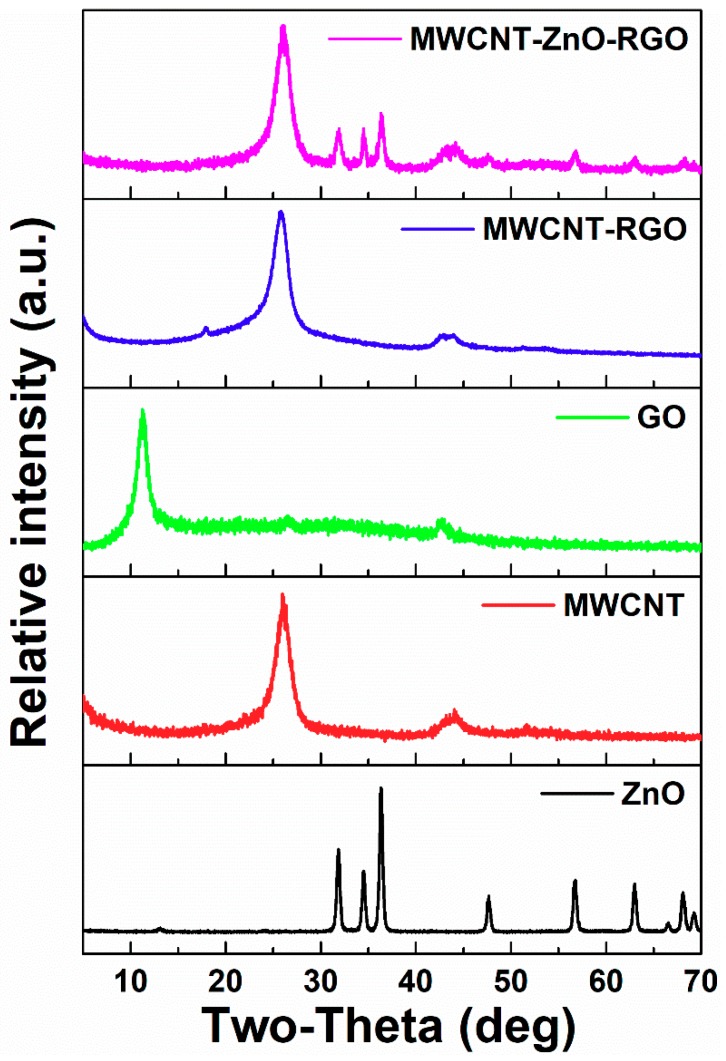
X-ray diffraction (XRD) patterns of ZnO, MWCNTs, GO, MWCNT–RGO, and MWCNT–ZnO–RGO powders. a.u. = arbitrary units.

Figure 2 shows the XRD of the ZnO, MWCNTs, GO, MWCNTs-RGO and MWCNT–ZnO–RGO powders, respectively. The diffraction peaks of MWCNT–ZnO–RGO hybrid nanoparticles can be well indexed to the characteristic diffraction peaks of hexagonal ZnO (Joint Committee on Powder Diffraction Standards, JCPDS#36-1451), which confirmed that the hexagonal structure of ZnO has successfully joined onto the hybrid during the hydrothermal process. According to the Scherrer formula, D = Kλ/Bcos *θ*, where K is 0.89, *θ* is the diffraction angle, and λ is the X-ray wavelength (0.154 nm). Taking the strongest diffraction peak 2*θ* = 36.32 to calculate D, the crystallite size is 23.78 nm. Also, a diffraction peak at 26.2° appears in MWCNT–RGO and MWCNT–ZnO–RGO, which is most likely from the CNTs [37]. The characteristic diffraction peak of GO shows a sharp peak at 2*θ* = 10.8°, corresponding to a layer distance (d-spacing) of about 0.82 nm. However, in comparison with GO, after hydrazine reduction of GO, no characteristic peak of GO is observed in MWCNT–RGO and MWCNT–ZnO–RGO hybrid particles, proving that GO is fully exfoliated and there are no restacked as-reduced graphene sheets existing in the two hybrid nanoparticles. [38].

**Figure 3:**
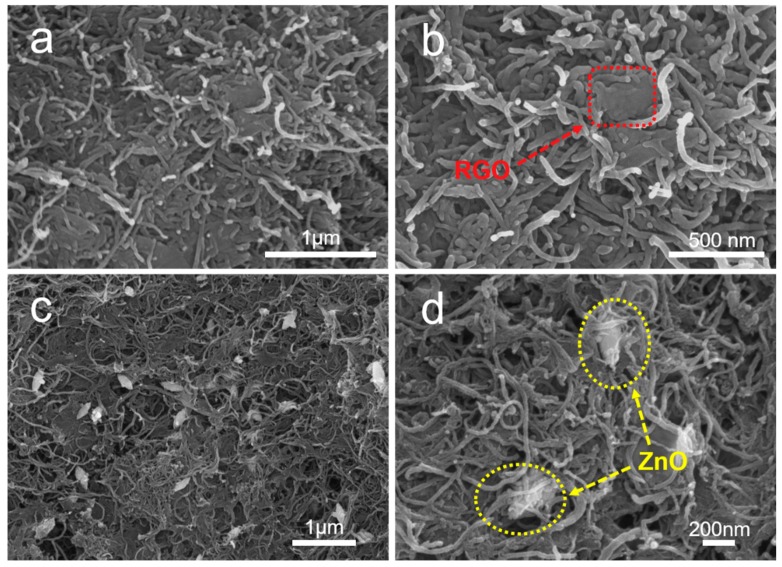
Scanning electron microscopy (SEM) images of the MWCNT–RGO hybrid particles (**a**,**b**) and MWCNT–ZnO–RGO hybrid particles (**c**,**d**). The RGO sheets are wrapped by the MWCNTs, and the ZnO nanorods are uniformly dispersed in MWCNTs.

The surface morphologies and microstructures of MWCNT–RGO hybrid particles and MWCNT–ZnO–RGO hybrid particles are investigated and observed by SEM. Figure 3a,b reveals that the MWCNTs are intertwined and covered with RGO sheets to assemble a 3D net structure. Furthermore, the RGO sheets labeled by the red dashed block in Figure 3b are embraced by the MWCNTs in the MWCNT–RGO hybrid particles, and the size of the RGO is about a few microns. In Figure 3c,d, we can observe that the single ZnO nanorods are uniformly distributed in the 3D net structure of the MWCNT–ZnO–RGO hybrid particles with an average length and width of 300 ± 10 nm and 100 ± 5 nm, respectively. This is larger compared with the particle size calculated from XRD, probably due to the carbon nanotubes wrapped around the surface of the ZnO nanorods. Also, after magnification, as shown in the yellow dashed circle (Figure 3d), the intimate contact among ZnO, RGO and MWCNTs indicates that the three components have strong interface interactions with each other. Therefore, compared with MWCNT–RGO, in MWCNT–ZnO–RGO hybrid particles, single ZnO nanorods can not only limit the neighboring MWCNTs agglomerating, but also the RGO sheets appear more wrinkled, which is more effective to enhance the interfacial interaction between the PVC matrix and the MWCNT–ZnO–RGO filler nanoparticles, thereby improving the mechanical properties of the polymer composites.

**Figure 4:**
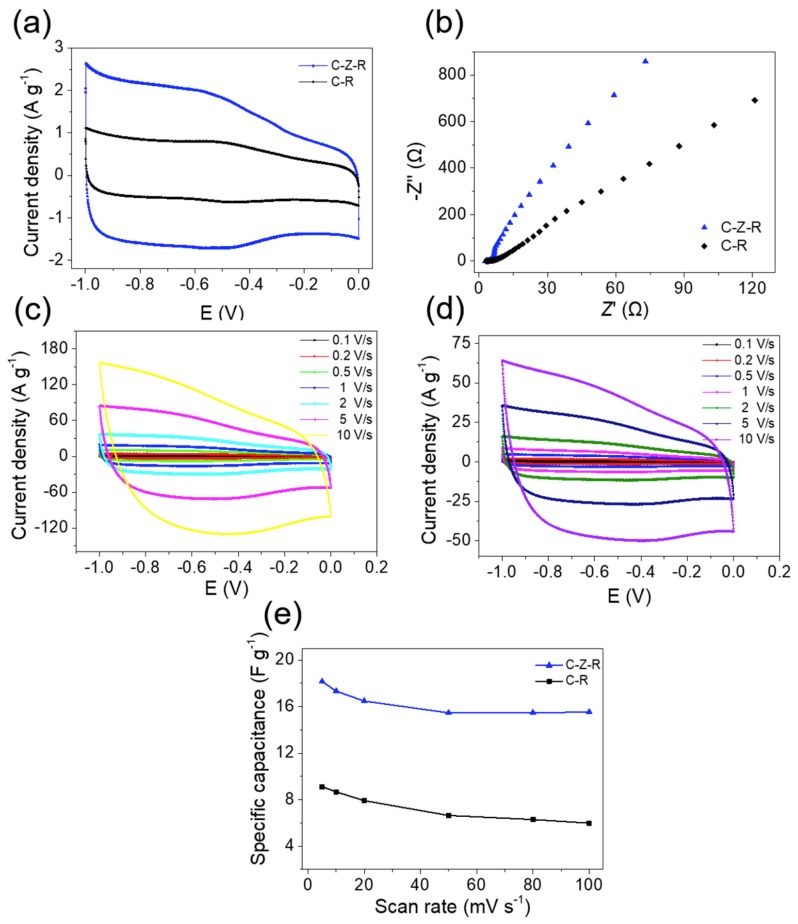
(**a**) Cyclic voltammetry (CV) curves of MWCNT–ZnO–RGO and MWCNT–RGO electrodes at the scan rate of 100 mV/s in 2M KOH. (**b**) Nyquist plots of MWCNT–ZnO–RGO and MWCNT–RGO. (**c**) CV curves of the MWCNT–ZnO–RGO electrode at different scan rates. (**d**) CV curves of the MWCNT–RGO electrode at different scan rates. (**e**) Mass specific capacitance of the MWCNT–ZnO–RGO and MWCNT–RGO electrode at different scan rates.

The interactions between ZnO nanorods, MWCNTs and RGO are further investigated by cyclic voltammetry (CV) examination. The electrochemical properties of the MWCNT–ZnO–RGO and MWCNT–RGO hybrid particles are first carried out in a three-electrode configuration in 2 M KOH aqueous solution (Figure 4a). In the literature, it is reported that the homogeneous distribution of single metal oxides among the RGO layers or MWCNTs will improve the specific capacitance of the whole electrode [39]. Figure 4a compares the CV curves of MWCNT–ZnO–RGO and MWCNT–RGO hybrid particles. At a high scan rate of 100 mV/s, we can see all the specimens exhibit symmetric charge and a nearly rectangular shape. Even at the scan rate of 10 V/s, they still keep their shapes very well (Figure 4c,d). This manifests as an excellent electrical double layer (EDL) in the electrode. Moreover, the different specific capacitances with different voltage sweep rates (5 mV/s, 10 mV/s, 20 mV/s, 30 mV/s, 50 mV/s, 80 mV/s, and 100 mV/s) are examined for MWCNT–ZnO–RGO and MWCNT–RGO hybrid particles. Obviously, the mass specific capacitance (*CSP*, *m*) of MWCNT–ZnO–RGO is better than the MWCNT–RGO nanofiller mass specific capacitance at all scan rates. The mass specific capacitance of all active materials, calculated from the CV curves in a three-electrode cell, is obtained from the following formula:

(1) $$Csp,m=\frac{Q}{2Um}=\frac{1}{2Uvm}\int_{U-}^{U+}i(U)dU\;$$

where m represents the mass of the active substances used in electrochemical testing; U (U = U + -U-), *Q*, *i(U)* and $Cs,electrode=\frac{2It}{U}$ represent the scanned potential window, total voltametric charge, current and the scan rate of the CV curve, respectively. In our research, the scanned potential window is 1 V, and *Q* is calculated by integrating positive and negative sweeps of current in the three-electrode cell of the CV curve. Moreover, the mass specific capacitance of MWCNT–ZnO–RGO hybrid particles, obtained from the CV curve in Figure 4a, is almost 2.6 times higher than MWCNT–RGO hybrid particles. The phenomenon may ascribe to such a fact that the introduction of ZnO greatly increases the pore structure, resulting in a high specific surface area of the MWCNT–ZnO–RGO hybrid particles.

In order to verify the existence of ZnO in favor of increasing the electron and ion transport of MWCNT–ZnO–RGO compared to MWCNT–RGO hybrid particles, we carried out electrochemical impedance spectroscopy (EIS) (Figure 4e) which measured electrolytes with the frequency ranging from 0.01 to 1 × 10^6^ Hz at 5 mV open circuit potential in the three-electrode cell in 2 M KOH aqueous solution. From the figure, it is easy to see that the two electrode materials have no semicircle in the high-frequency region. However, in the low-frequency region, MWCNT–ZnO–RGO hybrid particles exhibit a straight line almost vertical to the real axis, suggesting an ideal capacitive behavior of the MWCNT–ZnO–RGO electrode [40]. From the above results, there is a positive synergistic effect among ZnO, RGO and MWCNTs, indicating that there is a strong interaction between the three components. Previous studies have shown that filler particles, containing one component which has strong interfacial interaction with polymer molecular chains, leads to an enhancement of the mechanical properties [32,33,34,39].

### 3.2. Interfacial Interaction in the PVC/MWCNT–ZnO–RGO Nanocomposites

**Figure 5:**
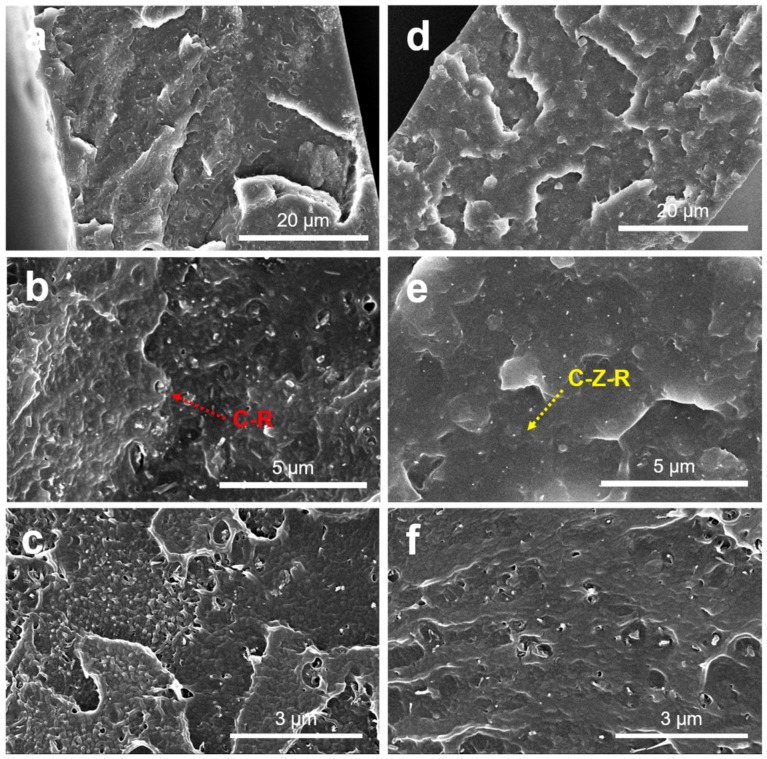
SEM pictures of the brittle fracture face of the PVC/2.0 wt.% MWCNT–RGO nanocomposites (**a**–**c**) and the PVC/2.0 wt.% MWCNT–ZnO–RGO nanocomposites (**d**–**f**).

The dispersion and distribution of fillers in the polymer matrix, as well as their interfacial bonding, are the two key factors in influencing the high macroscopic properties of the nanocomposites. The fracture morphology of PVC/2.0 wt.% CNT–ZnO–RGO composites and PVC/2.0 wt.% CNT–RGO composites are presented in Figure 5, which shows the dispersion state of CNT–RGO and CNT–ZnO–RGO in the PVC matrix. The CNT–RGO particles aggregate in the PVC matrix, as shown in Figure 5a–c. The CNT–ZnO–RGO hybrid particles are almost individual and uniformly distributed in the PVC composite films (Figure 5d–f), which makes a clear demonstration that ZnO may reduce the agglomeration of hybrid particles. It can also estimate the interfacial interactions from the SEM pictures between the hybrid particles and the PVC matrix. We can observe the gaps between the CNT–RGO particles and the PVC matrix, which means that the CNT–RGO particles have poor interaction with PVC therein. However, strong interactions detected between CNT–ZnO–RGO particles and PVC is ascribed to the ZnO having electrostatic/hydrogen bonding interactions with PVC chains. We can deduce that the PVC/RGO–ZnO–MWCNT composites exhibit excellent mechanical and conductive properties.

We can further prove the strong interfacial interactions between the MWCNT–ZnO–RGO filler and the PVC matrix by thermodynamic study, which calculates the spreading coefficient (*S_a−b_*, mN/m), which represents component a over component b from Equation (2) [41].

(2) $$S_{a-b}=\gamma_{b}-\gamma_{a}-\gamma_{ab}\;$$

where *γ_a_*, *γ_b_* and *γ_ab_* represent the surface tensions of component a, surface tensions of component b and the interfacial tension between *a* and *b*, respectively. If *S_a–b_* is positive, it represents that component b can be wrapped up by component *a*. If the opposite is true, it will not. *γ_a_* and *γ_b_* can be obtained from Equation (2). *γ_a_* contains the dispersive and polar components of *a*, and *γ_b_* contains the dispersive and polar components of *b*, which can be obtained from the following Equation (3).

(3) $$\left(1+\cos\theta \right)\gamma_{l}=2\left(\sqrt{\gamma_{s}^{d}\gamma_{l}^{d}}+\sqrt{\gamma_{s}^{p}\gamma_{l}^{p}}\right)\;$$

where *θ*, *γ^d^* and *γ^p^* are the contact angle between a pure liquid (l) and a solid (s), dispersive and polar components of the surface tension of component *a* and *b*, respectively. The interfacial tensions are obtained from the following harmonic-mean (Equation (4)) and geometric-mean (Equation (5)) equations [42,43]. Moreover, the spreading coefficients, evaluated from interfacial tensions, of MWCNT–ZnO–RGO and MWCNT–RGO samples are listed in Table 2. The spreading coefficients of PVC/MWCNT–ZnO–RGO composites, contrary to PVC/MWCNT–RGO, are all positive. This indicates that PVC could spread over the MWCNT–ZnO–RGO hybrid fillers. In addition, the results coincide with the conclusions we see in the above SEM pictures.

(4) $$\gamma_{12}=\gamma_{1}+\gamma_{2}-4\left(\frac{\gamma_{1}^{d}\gamma_{2}^{d}}{\gamma_{1}^{d}+\gamma_{2}^{d}}+\frac{\gamma_{1}^{p}\gamma_{2}^{p}}{\gamma_{1}^{p}+\gamma_{2}^{p}}\right),$$

(5) $$\gamma_{12}=\gamma_{1}+\gamma_{2}-2\left(\sqrt{\gamma_{1}^{d}\gamma_{2}^{d}}+\sqrt{\gamma_{1}^{p}\gamma_{2}^{p}}\right)\;$$

**Figure 6:**
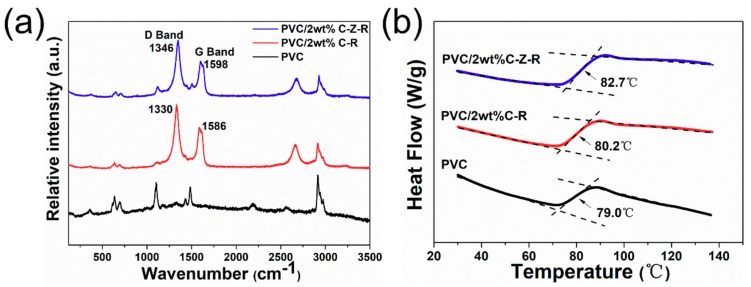
The Raman spectra (**a**) and the glass transition temperature (**b**) of the PVC, the PVC/2.0 wt.% MWCNT–ZnO–RGO composites and the PVC/2.0 wt.% MWCNT–RGO composites.

Raman spectroscopy is used to demonstrate the interactions on the molecular structure of an element in the composites. Figure 6a shows the Raman spectra for the PVC, the PVC/2.0 wt.% MWCNT–ZnO–RGO composites and the PVC/2.0 wt.% MWCNT–RGO composites. The main characteristic peak positions of the Raman spectroscopy in PVC are as follows: the band structure of C–Cl stretching, the C–C stretching vibration double bonds, the CH_2_ twist–CH_2_ wag vibrational and CH_2_ bending come out at 600–700 cm^−1^, 1127 cm^−1^ and 1515 cm^−1^, 1335 cm^−1^ and 1432 cm^−1^, respectively [44].

The D band and G band are shown in the Raman spectra of the PVC/2.0 wt.% MWCNT–ZnO–RGO and the PVC/2.0 wt.% MWCNT–RGO composites. The D band and G band are attributed to the vibration of disordered sp^2^-bonded carbon atoms and the vibration of sp^2^-bond carbon atoms in the two-dimensional hexagonal lattice, respectively. The C–C stretching vibration double bonds appeared at around 1127 cm^−1^ and 1515 cm^−1^. With the addition of ZnO, the maximum in the D band peak is moved by 16 cm^−1^ and the G band peak is moved by 12 cm^−1^. The shifting of the D and G band peaks can be accounted for by the existence of ZnO nanorods that exhibit electrostatic/hydrogen bonding with PVC chains and dispersion in the PVC matrix as a result of polymer penetration into the MWCNT–ZnO–RGO hybrid particles during mixing [45].

The interface interaction between the polymer matrix and the filler in the polymer nanocomposites can be further studied from the glass transition temperature (Tg). Figure 6b reveals that the corresponding Tg of pure PVC, PVC/2.0 wt.% RGO–MWCNT composites and PVC/2.0 wt.% RGO–ZnO–MWCNT composites are 79.0 °C, 80.2 °C and 82.7 °C, respectively. The Tg of these two composites are both higher than the pure PVC polymer, which is perhaps ascribed to the enhancement of the interfacial interaction between the PVC molecular chains and the two different nanofillers. Furthermore, the glass transition temperature of PVC/2.0 wt.% RGO–ZnO–MWCNT composites is 2.5 °C higher than PVC/2.0 wt.% RGO–MWCNT composites at the same content of particles. This indicates that the presence of ZnO nanorods could further restrict the chain segment mobility of PVC.

**Figure 7:**
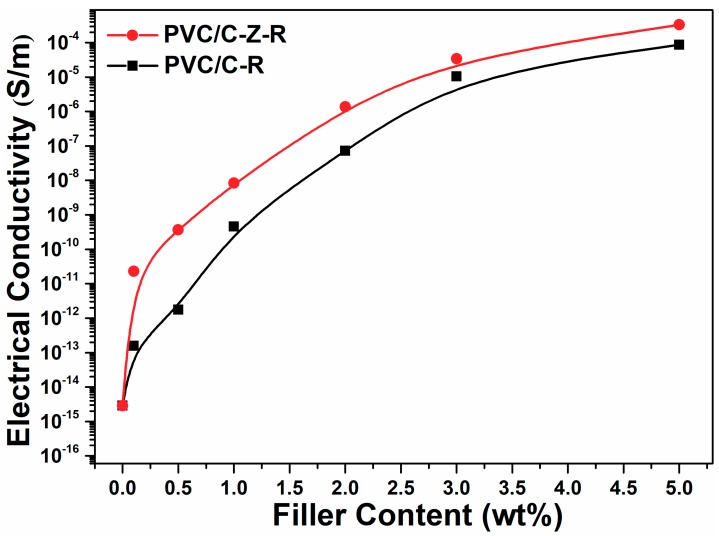
The electrical conductivity of composites varied by filler content. The content of MWCNT–RGO and MWCNT–ZnO–RGO particles are varied from 0 wt.%, 0.1 wt.%, 0.5 wt.%, 1.0 wt.%, 2.0 wt.%, 3.0 wt.% and 5.0 wt.% in PVC.

The electrical conductivity of the composites is plotted as a function of RGO–MWCNT and RGO–ZnO–MWCNT content in Figure 7. This figure shows typical percolation behavior: the approximate percolation thresholds are found to be around 1.0 wt.% filler content. It is usually considered that a conductive network exists in PVC/RGO–MWCNT and PVC/RGO–ZnO–MWCNT composites in which the fillers form a conductive path when the filler loading reaches the conductivity threshold. The incorporation of 5.0 wt.% RGO–MWCNT and RGO–ZnO–MWCNT increased the electric conductivity of the composites to 8.72 × 10^−5^ S/m and 3.32 × 10^−4^ S/m—about eleven orders of magnitude compared to the pure PVC (2.89 × 10^−15^ S/m). However, the composites containing RGO–ZnO–MWCNT exhibit a more conspicuous enhancement in electrical conductivity than their counterparts containing RGO–MWCNT, especially if the filler content is below 1.0 wt.%. At the same content (0.1 wt.% and 0.5 wt.%), the electric conductivity of the PVC/RGO–ZnO–MWCNT composites increased to 2.31 × 10^−11^ S/m and 3.68 × 10^−10^ S/m, while the electric conductivity of the PVC/RGO–MWCNT composites increased to 1.57 × 10^−13^ S/m and 1.78 × 10^−12^ S/m, showing the advantage of ZnO nanorods acting as efficient joint points to enhance the conductivity of the nanocomposites.

**Figure 8:**
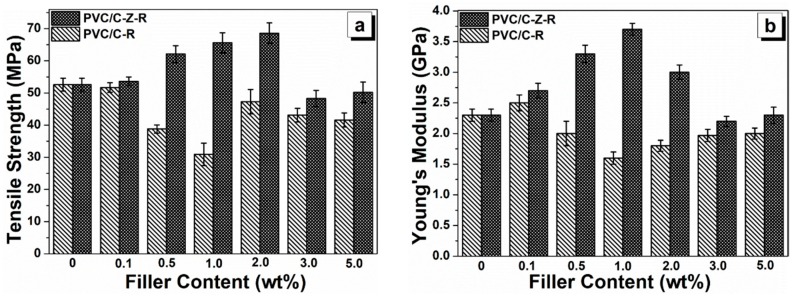
The tensile strength (**a**) and Young’s modulus (**b**) of the PVC composite films with different particles. The content of MWCNT–RGO and MWCNT–ZnO–RGO particles are varied from 0 wt.%, 0.1 wt.%, 0.5 wt.%, 1.0 wt.%, 2.0 wt.%, 3.0 wt.% and 5.0 wt.% in PVC.

The dispersion of fillers in the matrix and their interactions affect the nano- and micro-structures in the polymer composite, thereby affecting the composite’s mechanical properties. The tensile strength (Figure 8a) and Young’s modulus (Figure 8b) of the PVC composites with different RGO–MWCNT and RGO–ZnO–MWCNT filler contents ranging from 0.1 wt.% to 5.0 wt.% are shown. Obviously, incorporation of low content RGO–ZnO–MWCNT hybrid particles, i.e., not more than 2.0 wt.%, enhances both the tensile strength and Young’s modulus in the PVC composites. As the RGO–ZnO–MWCNT hybrid particle loading reaches 2.0 wt.%, the tensile strength reaches the maximum value of 68.6 MPa, which is enhanced by 30.8% compared to the unfilled PVC (52.6 MPa). On the contrary, the tensile strength is reduced by 41.3% (30.9 MPa) after the addition of 1.0 wt.% RGO–MWCNT fillers to the PVC matrix. Moreover, the incorporation of RGO–ZnO–MWCNT hybrid particles exhibits a great effect on the Young’s modulus of PVC. In the range of 0.1–1.0 wt.% of RGO–ZnO–MWCNT fillers, composites show a continuous increase in Young’s modulus with the increase in filler amount. The highest value is achieved with 1.0 wt.% RGO–ZnO–MWCNT fillers (3.7 GPa), which is increased by 60.9% compared to the value of the unfilled PVC (2.3 GPa). However, the Young’s modulus of the PVC/RGO–MWCNT composites is decreased with an increased loading of RGO–MWCNT particles up to 5.0 wt.%, and the minimum value at 1.0 wt.% is increased by 30.4% compared to pure PVC.

**Figure 9:**
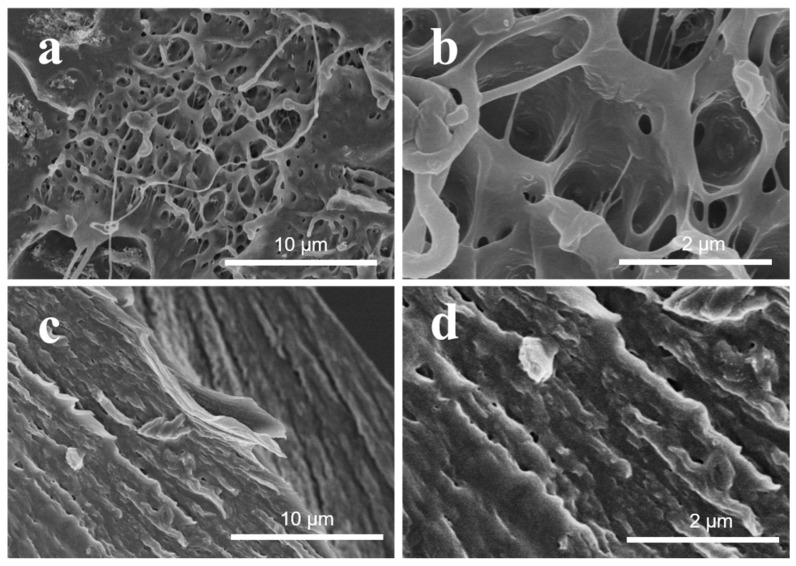
SEM pictures of the tensile fractured surface of the PVC/2.0 wt.% MWCNT–RGO particles (**a**,**b**) and the PVC/2.0 wt.% MWCNT–ZnO–RGO nanocomposites (**c**,**d**).

To reveal the reasons for the reinforcing mechanism, the fractured surface of PVC/2.0 wt.% RGO–MWCNT and PVC/2.0 wt.% RGO–ZnO–MWCNT composites after tensile testing are investigated by SEM. As shown in Figure 9a,b, the fractured faces of the PVC/RGO–MWCNT composites are rough and have many cavities. These results indicate that the PVC/RGO–MWCNT composites are prone to semi-brittle failure and have a weak interfacial interaction between RGO–MWCNT nanoparticles and PVC molecular chains. Nevertheless, compared with the PVC/RGO–MWCNT composites, Figure 9b,c revealed that the fractured faces of PVC/RGO–ZnO–MWCNT composites are more smooth and flat. At the beginning of the stretching process in the neck of the samples, the stress causes the debonded particles to locally increase at the particle–polymer interface. For further stretching, these debonded areas would form many holes on account of bad interactions with the polymer matrix [46]. It can be seen that many voids developed in the course of the stretching in Figure 9a,b. In contrast, the tensile fractured surface of PVC/RGO–ZnO–MWCNT composites showed fewer defects. These consequences demonstrate that the RGO–ZnO–MWCNT hybrid particles have tough interfacial interactions with the PVC molecular chains, whereas there were poor interfacial interactions between the RGO–MWCNT nanoparticles and the PVC molecular chains.

The improvement of the mechanical properties is accounted for by the better interfacial adhesion of PVC and RGO–ZnO–MWCNT hybrid fillers. The presence of ZnO nanorods acts to afford stable interphase interactions that are dominated by electrostatic interactions and p–π stacking interactions between RGO and MWCNTs and hydrogen bonding and electrostatic interactions with the PVC molecular chains. These Zn^2+^ ions absorb onto the external surface of RGO through electrostatic interactions and p–π stacking interactions to generate stable RGO–ZnO–MWCNT particles. The strong dipole attraction of ZnO is beneficial for hydrogen bonding and electrostatic interaction with PVC molecular chains. Therefore, ZnO nanorods could act as a “joint” to connect the RGO–ZnO–MWCNT and PVC molecular chains. With regard to the PVC/RGO–MWCNT composites, the RGO–MWCNT particles have no interaction with PVC molecular chains, resulting in weak interactions with PVC and agglomerations in the PVC matrix.

## 4. Conclusions

In summary, a facile in-situ method is established for ZnO nanorod decoration onto RGO–MWCNT hybrids by utilizing ZnO as a bridge to connect RGO–MWCNT fillers and PVC chains. We have prepared PVC composites containing RGO–ZnO–MWCNT hybrid particles. The ZnO nanorods interact strongly with MWCNTs and RGO through p–π stacking/electrostatic interactions without destroying their own structure. Moreover, the ZnO nanorods uniformly distribute in a three-dimensional (3D) network which consists of the one-dimensional structure of MWCNTs and two-dimensional structure of RGO, similar to the “joints” in fishing nets. The tensile strength and Young’s modulus of PVC increase by 30.8% (68.6 MPa) and 60.9% (3.7 GPa) when adding 2.0 wt.% and 1.0 wt.% RGO–ZnO–MWCNT hybrid particles, respectively. The electrical conductivity of composites with RGO–ZnO–MWCNT hybrid particles is significantly higher than those containing RGO–MWCNT hybrid particles, especially if the filler content is below 1.0 wt.%. The improvement in electrical conductivity and mechanical properties is basically ascribed to the presence of ZnO. The Zn^2+^ ions have electrostatic interactions and p–π stacking interactions with RGO and MWCNTs to generate the steady RGO–ZnO–MWCNT hybrid particles. The hydrogen bonds and electrostatic attraction interactions exist between the ZnO nanorods and the PVC molecular chains.

## Figures and Tables

**Table 1:** Summary of all investigated nanocomposite configurations.

Samples	Percentage of Filler Loading in PVC (wt.%)	Abbreviation
PVC	0	PVC
MWCNT–RGO	0	C–R
MWCNT–ZnO–RGO	0	C–Z–R
PVC/0.1 wt.% MWCNT–RGO	MWCNT–RGO = 0.1 wt.%	PVC/0.1 wt.% C–R
PVC/0.2 wt.% MWCNT–RGO	MWCNT–RGO = 0.2 wt.%	PVC/0.2 wt.% C–R
PVC/0.5 wt.% MWCNT–RGO	MWCNT–RGO = 0.5 wt.%	PVC/0.5 wt.% C–R
PVC/1.0 wt.% MWCNT–RGO	MWCNT–RGO = 1.0 wt.%	PVC/1.0 wt.% C–R
PVC/2.0 wt.% MWCNT–RGO	MWCNT–RGO = 2.0 wt.%	PVC/2.0 wt.% C–R
PVC/3.0 wt.% MWCNT–RGO	MWCNT–RGO = 3.0 wt.%	PVC/3.0 wt.% C–R
PVC/5.0 wt.% MWCNT–RGO	MWCNT–RGO = 5.0 wt.%	PVC/5.0 wt.% C–R
PVC/0.1 wt.% MWCNT–ZnO–RGO	MWCNT–ZnO–RGO = 0.1 wt.%	PVC/0.1 wt.% C–Z–R
PVC/0.2 wt.% MWCNT–ZnO–RGO	MWCNT–ZnO–RGO = 0.2 wt.%	PVC/0.2 wt.% C–Z–R
PVC/0.5 wt.% MWCNT–ZnO–RGO	MWCNT–ZnO–RGO = 0.5 wt.%	PVC/0.5 wt.% C–Z–R
PVC/1.0 wt.% MWCNT–ZnO–RGO	MWCNT–ZnO–RGO = 1.0 wt.%	PVC/1.0 wt.% C–Z–R
PVC/2.0 wt.% MWCNT–ZnO–RGO	MWCNT–ZnO–RGO = 2.0 wt.%	PVC/2.0 wt.% C–Z–R
PVC/3.0 wt.% MWCNT–ZnO–RGO	MWCNT–ZnO–RGO = 3.0 wt.%	PVC/3.0 wt.% C–Z–R
PVC/5.0 wt.% MWCNT–ZnO–RGO	MWCNT–ZnO–RGO = 5.0 wt.%	PVC/5.0 wt.% C–Z–R

The weight ratio of MWCNT:RGO = 3:1 and MWCNT:RGO:ZnO = 3:1:0.27. wt.% = weight percent.

**Table 2:** Surface tension, interfacial energy and spreading coefficient results of PVC, MWCNT–ZnO–RGO, and MWCNT–RGO.

Samples	Surface Tension (mN/m)			Interfacial Energies with PVC (mN/m)		Spreading Coefficient with PVC (mN/m)	
	Total (γ)	Dispersive $\mathrm{Part}\;(\gamma^{d})$	Polar Part $\left(\gamma^{p}\right)$	Harmonic	Geometric	Harmonic	Geometric
PVC	43.2	42.0	1.2				
MWCNT–RGO	71.2	69.04	2.16	6.86	50.76	−34.86	−78.75
MWCNT–ZnO–RGO	25.72	25.5	0.22	4.74	2.45	12.74	15.03
